# Associations between post-acute sequelae of SARS-CoV-2, COVID-19 vaccination and HIV infection: a United States cohort study

**DOI:** 10.3389/fimmu.2024.1297195

**Published:** 2024-01-22

**Authors:** George A. Yendewa, Jaime Abraham Perez, Nirav Patil, Grace A. McComsey

**Affiliations:** ^1^ Department of Medicine, Case Western Reserve University School of Medicine, Cleveland, OH, United States; ^2^ Division of Infectious Diseases and HIV Medicine, University Hospitals Cleveland Medical Center, Cleveland, OH, United States; ^3^ Center for Clinical Research, University Hospitals Cleveland Medical Center, Cleveland, OH, United States

**Keywords:** HIV, COVID-19, PASC, vaccination, comorbidities

## Abstract

**Background:**

People with HIV (PWH) are at higher risk of complications from acute COVID-19, but their risk of subsequent post-acute sequelae of SARS-CoV2 (PASC) remains unclear. Although vaccination is protective of PASC among survivors in the general population, its effectiveness in PWH has not been explored.

**Methods:**

We used the TriNetX health research database to identify patients with and without HIV aged ≥18 years with confirmed SARS-CoV-2 between January 1, 2020 and July 20, 2023. We employed 1:1 propensity score matching to balance HIV and non-HIV cohorts based on demographics and key comorbidities. The primary outcomes accessed odds of PASC and mortality and secondary outcomes assessed odds of PASC and mortality by vaccination status. PASC was defined as new-onset conditions ≥ 28 days after COVID-19 diagnosis. We reported odd ratios (OR) of outcomes with 95% confidence intervals (CI), with statistical significance set at p < 0.05.

**Results:**

Of 3,029,340 people with confirmed SARS-CoV-2 infection, 0.5% (n=13,214) were PWH, with 7.5% of PWH (n=989) vaccinated. After 28 days post-COVID-19, PWH had higher odds of mortality compared with their non-HIV counterparts (OR 1.22, 95% CI 1.06-1.40) and developing new-onset HTN (OR 1.18, 95% CI 1.03-1.36), heart disease (OR 1.35 95% CI 1.18-1.54), malignancy (OR 1.49, 95% CI 1.22-1.81), and mental disorders (OR 1.62, 95% CI 1.42-1.85). Furthermore, vaccinated PWH had significantly lower odds of death (OR 0.63, 95% CI 0.42-0.93) and new-onset PASC outcomes: DM (OR 0.65, 95% CI 0.43-0.99), heart disease (OR 0.58, 95% CI 0.4-0.85), mental disorders (OR 0.66, 95% CI 0.43-1.00), fatigue (OR 0.82, 95% CI 0.67-0.98), respiratory (OR 0.82, 95% CI 0.70-0.95) and gastrointestinal symptoms (OR 0.78, 95% CI 0.67-0.90).

**Conclusion:**

HIV-positive status increased PASC odds, while COVID-19 vaccination reduced PASC and all-cause mortality risks in PWH.

## 1 Introduction

The coronavirus disease 2019 (COVID-19) pandemic, caused by the severe acute respiratory syndrome coronavirus (SARS-CoV-2), remains a major global challenge, resulting in millions of confirmed cases and attributable deaths (1). Following the acute phase of SARS-CoV-2 infection, a significant proportion of survivors experience a constellation of persistent symptoms or new-onset health conditions collectively referred to as the post-acute sequelae of SARS-CoV-2 (PASC) or long COVID (2, 3). The true prevalence of PASC remains unknown; however, systematic reviews and meta-analyses of studies from the United States and globally suggest that 6% to 90% of individuals diagnosed with COVID-19 experience one or more PASC conditions (4–6). Frequently described manifestations of PASC include persistence after the acute illness of fatigue, malaise, myalgias, joint pains, loss of taste or smell, as well as cardiovascular, pulmonary, gastrointestinal, endocrine, mental health, and neurocognitive disorders (2, 3, 7–9–). These sequelae may follow asymptomatic, mild, or severe infection and may persist for many months, resulting in significant functional disability and reduction in the quality of life of survivors (2, 8–11).

The risk factors associated with PASC are incompletely understood; however, current evidence suggests that the occurrence and severity of PASC are influenced by the intensity of the initial acute illness (12), virus-specific factors such as the type of SARS-CoV-2 variant (13, 14), and the presence of premorbid risk factors, including older age, higher body mass index (BMI), cardiovascular disease, diabetes mellitus (DM), allergies, chronic lung disease, chronic kidney disease, and malignancy (15). People with HIV (PWH) have more severe COVID-19 symptoms and adverse outcomes (16) and HIV infection has been suggested as a potential risk factor for PASC; however, there is a dearth of supporting evidence to confirm this. Compared with the general population, people with HIV (PWH) tend to have a higher burden of comorbidities and predisposing risk factors at baseline (17, 18). Additionally, PWH are known to experience chronic residual systemic inflammation, immune activation, and antiretroviral treatment (ART)-induced mitochondrial toxicities even in well-treated HIV disease, which may amplify the “cytokine storm” in COVID-19 and increase the risk of PASC in PWH (17, 18). In a recent study by Peluso and colleagues (19), PWH recovering from COVID-19 had a 4-fold higher odds of developing PASC symptoms compared with their non-HIV counterparts. However, the study enrolled a small sample size (n=39 PWH and n=43 without HIV) and relied solely on persistent symptoms for the definition of PASC (19), thus warranting the need for larger and more systematic studies to examine the association between HIV status and the risk of PASC.

Due to the heterogeneity of the health conditions involved, current guidelines emphasize adopting a holistic and multidisciplinary approach in the management of PASC (20, 21). As the COVID-19 pandemic continues and the number of survivors increases, this is projected to incur considerable healthcare costs and loss in productivity for years to come (22). From a risk mitigation perspective, preventing COVID-19 infection is the most effective means of preventing PASC occurrence. Vaccination against COVID-19 is effective at preventing infection (23, 24) and reducing the severity of acute illness (15) and remains a priority public health intervention in controlling the pandemic. In a recent study, we showed that COVID-19 vaccination reduces the risk of PASC symptoms, new-onset health conditions, and all-cause mortality (25). However, no large-scale studies have systematically assessed the risk of PASC or the impact of vaccination on its occurrence among PWH.

The primary objectives of this study were to describe the association between HIV status and the risk of PASC and all-cause mortality by comparing PWH and their non-HIV counterparts in a large cohort in the United States. Additionally, as secondary objectives, we further assessed the effect of COVID-19 vaccination on the risk of PASC and all-cause mortality among vaccinated and unvaccinated PWH. Lastly, within the PWH population, we investigated the association between HIV disease indices (CD4 cell count, HIV viral load, and ART) and PASC.

## 2 Materials and methods

### 2.1 Data source

We used a large national health research network with data sourced from 69 health care organizations (HCO) within the United States (TriNetX, a global federated health research network with waiver from WCG IRB). We included any adult aged ≥18 years with a HCO encounter between January 1, 2020 and July 20, 2023 (last date of data access). TriNetX continuously aggregates clinical data directly from the electronic health records (EHRs) of participating HCOs. TriNetX provides de-identified data, transformed into a proprietary data schema, including an extensive data quality and accuracy assessment. TriNetX does not provide any identifiers on participating HCOs; however, a typical participating HCO includes a large academic health center with inpatient, outpatient, and specialty care services.

### 2.2 Patient selection, definitions and study outcomes

We first identified all adult patients with positive SARS-CoV-2 infection (COVID-19 International Classification of Diseases (ICD) 10th Revision codes, ICD-10: U07.1, J12.82, U07.2, or positive SARS-CoV-2 and related RNA test or positive Rapid Antigen test). We further categorized patient cohorts into those with previous HIV diagnosis (ICD-10: B20), and those without HIV. For all cohorts, we collected clinical data including patient demographics (age, sex, race and ethnicity), pre-existing comorbid conditions regarded as risk factors for COVID-19 severity (overweight and obesity, neoplasms, hypertension (HTN), heart disease, DM, chronic kidney disease, chronic obstructive lung disease and transplanted organ) COVID-19 vaccination status, and medication history. In addition, for PWH, we collected information on CD4 count, viral load (HIV-1 RNA) and antiretroviral treatment (ART).

The hypothesis of this study was that PWH with a history of COVID-19 are at higher risk of PASC when compared to propensity-matched population of non-HIV COVID-19 survivors. The primary outcomes were the odds of PASC and all-cause mortality compared by HIV status. The secondary outcomes were the odds of PASC and mortality among PWH, compared by COVID-19 vaccination history. PASC was defined using the criteria proposed by the United States Center for Disease Control and Prevention (CDC) as either the persistence of COVID-attributable symptoms or the occurrence of new-onset health conditions at least 28 days following the first COVID-19 diagnosis (26). We used the 28-day cutoff after the COVID-19 diagnosis to ensure that the PASC diagnosis captured did not include medical conditions patients might have had prior to their COVID-19 diagnosis.

For PASC symptoms, we selected the following commonly reported manifestations: respiratory symptoms, fatigue, headache, body ache, diarrhea/constipation and neurocognitive disfunction. Similarly, for new-onset conditions, we selected commonly reported outcomes, as follows: heart disease, HTN, DM, malignancy, thrombosis, thyroid disease, rheumatoid arthritis and mental disorders. We included all-cause mortality in all outcomes assessments as several PASC conditions (e.g., heart disease, thrombosis) are associated with high risk of mortality. All symptoms or conditions selected for inclusion in the study were based on healthcare provider entries of diagnoses and their corresponding International Classification of Diseases Tenth Revision (ICD-10) codes into the EHRs of patients. A full description of study definitions and variables used to query the TriNetX database and ICD-10 codes for specific diagnoses are provided in the **Supplementary Materials**.

Within the HIV cohort, we performed a secondary analysis to further assess outcomes based on degree of immunosuppression (CD4 < 200 cells/mm3 vs ≥ 200 cells/mm3), virologic suppression (HIV RNA < 200 copies/mL vs ≥ 200 copies/mL), ART class, and circulating SARS-CoV-2 variant. Since there was no information on SARS-CoV-2 variants in patient records, we used COVID-19 pandemic variant periods instead, as reported by the CDC (27): July 1, 2021 to November 30, 2021 for Delta variant and December 1, 2021 to July 20, 2023 for Omicron variant.

### 2.3 Statistical analyses

Characteristics of study cohorts were described using mean ± standard deviation for continuous variables and frequency and percentages for categorical variables. All analyses were conducted using TriNetX Advanced Analytics platform. To address potential confounders that could bias our results, cohorts were balanced using 1:1 greedy nearest-neighbor propensity score matching based on age, sex, race, ethnicity, BMI, and comorbid conditions (**Table 1**). For continuous data, we performed independent t-tests. For categorical data, we performed chi-square tests. For outcomes of interest, we calculated odds ratios (ORs) and 95% confidence intervals (CIs), with p < 0.05 considered statistically significant.

**Table 1:** Baseline characteristics between COVID-19 patients with or without a HIV positive diagnosis (HIV+, HIV-), both before and after propensity score matching.

	Before Matching			After Matching		
Characteristic Name	HIV +	HIV -	p-Value	HIV +	HIV -	p-Value
**N**	13214	3016126		13212	13212	
**Age**	49.38 ± 13.71	48.43 ± 18.2	<0.001	49.38 ± 13.71	49.5 ± 15.59	0.506
At least 50 years	6977 (52.8%)	1424498 (47.2%)	<0.001	6975 (52.8%)	6990 (52.9%)	0.853
At least 65 years	1773 (13.4%)	670155 (22.2%)	<0.001	1773 (13.4%)	1786 (13.5%)	0.815
BMI	28.58 ± 6.73	29.72 ± 7.04	<0.001	28.58 ± 6.74	29.84 ± 7.09	<0.001
At least 25 kg/m2	4075 (30.8%)	825539 (27.4%)	<0.001	2414 (34.2%)	28 (35.0%)	<0.001
At least 30 kg/m2	2538 (19.2%)	546423 (18.1%)	0.001	4073 (30.8%)	4061 (30.7%)	0.873
Sex						
Male	8989 (68.0%)	1253606 (41.6%)	<0.001	8987 (68.0%)	9018 (68.3%)	0.682
Female	4223 (32.0%)	1732998 (57.5%)	<0.001	4223 (32.0%)	4193 (31.7%)	0.692
Unknown	10 (0.1%)*	29522 (1.0%)	<0.001	10 (0.1%)*	10 (0.1%)*	1.000
Race						
Black or African American	5737 (43.4%)	502171 (16.7%)	<0.001	5735 (43.4%)	5735 (43.4%)	1.000
White	4783 (36.2%)	1790465 (59.4%)	<0.001	4783 (36.2%)	4778 (36.2%)	0.949
Unknown Race	2469 (18.7%)	633157 (21.0%)	<0.001	2469 (18.7%)	2455 (18.6%)	0.825
Asian	146 (1.1%)	72796 (2.4%)	<0.001	146 (1.1%)	155 (1.2%)	0.602
American Indian or Alaska Native	63 (0.5%)	12228 (0.4%)	0.198	63 (0.5%)	70 (0.5%)	0.543
Native Hawaiian or Other Pacific Islander	16 (0.1%)	5309 (0.2%)	0.133	16 (0.1%)	19 (0.1%)	0.612
Ethnicity						
Not Hispanic or Latino	8749 (66.2%)	1744596 (57.8%)	<0.001	8749 (66.2%)	8776 (66.4%)	0.725
Hispanic or Latino	1554 (11.8%)	250600 (8.3%)	<0.001	1552 (11.7%)	1535 (11.6%)	0.745
Unknown Ethnicity	2911 (22.0%)	1020930 (33.8%)	<0.001	2911 (22.0%)	2901 (22.0%)	0.882
Comorbid conditions						
Hypertensive diseases	6910 (52.3%)	890736 (29.5%)	<0.001	6908 (52.3%)	6952 (52.6%)	0.588
Neoplasms	4979 (37.7%)	618799 (20.5%)	<0.001	4977 (37.7%)	4958 (37.5%)	0.809
Hyperlipidemia	4517 (34.2%)	585172 (19.4%)	<0.001	4515 (34.2%)	4514 (34.2%)	0.990
Overweight and obesity	3693 (27.9%)	568424 (18.8%)	<0.001	3693 (28.0%)	3724 (28.2%)	0.671
Diabetes mellitus	3198 (24.2%)	418044 (13.9%)	<0.001	3196 (24.2%)	3213 (24.3%)	0.807
Chronic kidney disease	2540 (19.2%)	194677 (6.5%)	<0.001	2538 (19.2%)	2529 (19.1%)	0.888
Ischemic heart diseases	2245 (17.0%)	283916 (9.4%)	<0.001	2244 (17.0%)	2243 (17.0%)	0.987
COPD	1482 (11.2%)	145494 (4.8%)	<0.001	1481 (11.2%)	1455 (11.0%)	0.611
Organ Transplant History	458 (3.5%)	37406 (1.2%)	<0.001	458 (3.5%)	459 (3.5%)	0.973
Laboratory findings						
CD4,/mL	585 ± 383			585 ± 383		
At most 200/uL	2414 (34.2%)			2414 (34.2%)		
HIV 1 RNA (viral load)	17591 ± 95842			17591 ± 95842		
At most 50 {copies}/mL	1018 (77.7%)			1018 (77.7%)		
**COVID-19 vaccine**	989 (7.5%)	120460 (4.0%)	<0.001	989 (7.5%)	689 (5.2%)	0.093

*TriNetX automatically round patient counts of 1-9, up to 10.

### 2.4 Patient consent statement

The study was approved by the Institution Board Review committee at Case Western Reserve University/University Hospitals Cleveland Medical Center. Written informed consent was waived as data from the TriNetX system safeguards patient’s privacy by reporting deidentified data.

## 3 Results

### 3.1 Baseline characteristics of COVID-19 patients with and without a HIV diagnosis

**Table 1** reports the baseline characteristics by HIV status before and after propensity score matching. Of 3,029,340 people with confirmed SARS-CoV-2 infection, 0.5% (n=13,214) were PWH, with 7.5% of PWH (n=989) having documented evidence of having received at least 1 dose of any COVID-19 vaccine. Compared to their non-HIV counterparts, PWH were older (mean age 49.38 ± 13.71years vs 48.43 ± 18.2 years, p < 0.001), had fewer females (32.0% vs 57.5%, p < 0.001) and a higher proportion of African Americans (43.3% vs 16.7%, p < 0.001), and Hispanics (11.8% vs 8.3%, p < 0.001). At baseline, PWH were more likely to have an underlying comorbidity, including a history of HTN (52.3% vs 29.5%, p < 0.001), cancer (37.7% vs 20.5%, p < 0.001), DM (36·9% vs 13.7%, p < 0.001), overweight or obesity (36·9% vs 18·7%, p < 0.001), hyperlipidemia (34.2% vs 19.4%, p < 0.001), overweight/obesity (27.9% vs 18.8%, p < 0.001), DM (24.2% vs 13.9%, p < 0.001), chronic kidney disease (19.2% vs 6.5%, p < 0.001), and ischemic heart disease (17.0% vs 9.4%, p < 0.001). Of PWH with available data, the mean CD4 count was 584.54 ± 383 cells/mm3 and 77.7% were virally suppressed (HIV-1 RNA < 50 copies/mL).

### 3.2 PASC outcomes in patients with and without HIV diagnosis

**Table 2** displays the incidence of new-onset diagnosis or persistent symptoms captured at least 28 days after COVID-19 diagnosis. After propensity score matching, PWH had higher odds of death (OR 1.22, 95% CI 1.06-1.4; p = 0.006).The odds of developing new-onset HTN among PWH was 1.18 (95% CI 1.03, 1.36; p < 0.019), heart disease 1.35 (95% 1.18-1.54; p < 0.001), malignancy 1.49 (95% CI 1.22-1.81; p < 0.001), and mental disorders 1.62 (95% CI 1.42-1.85; p < 0.001). Similarly, the odds (OR) of persistent COVID-attributed symptoms remained significantly higher among PWH in a wide range organ systems: respiratory symptoms 1.41 (95% 1.33-1.50; p < 0.001), gastrointestinal symptoms 1.64 (95% CI 1.54-1.73; p < 0.001), headache 1.68 (95% CI 1.53-1.84; p < 0.001), fatigue 1.37 (95% CI, 1.26-1.49; p < 0·0001), body aches 1.41 (95% CI 1.25-1.6; p < 0.001), disturbances to smell and taste 1.43 (95%, 1-2.03; p = 0.049), cognitive impairment 1.43 (95%CI 1.11-1.86; p = 0.006), and abnormal movements 1.21 (95% CI 1.03, 1.43, p = 0.021). However, there was no significant association between odds of each new-onset PASC condition and CD4 count, HIV viremia or class of ART (**Supplementary Materials**).

**Table 2:** PASC related outcomes between COVID-19 patients with or without a HIV positive diagnosis (HIV+, HIV-) after propensity score matching.

Outcome	HIV+	HIV-	OR (95% CI)	p-Value
Mortality	441 (3.3%)	364 (2.8%)	1.22 (1.06, 1.40)	0.006
Hypertension	453 (7.3%)	383 (6.2%)	1.18 (1.03, 1.36)	0.019
Diabetes	333 (3.3%)	300 (3.0%)	1.11 (0.95, 1.3)	0.199
Thyroid Disorders	117 (1.0%)	110 (0.9%)	1.06 (0.82, 1.38)	0.646
Heart Disease	525 (6.0%)	415 (4.5%)	1.35 (1.18, 1.54)	<0.001
Malignancy	249 (2.2%)	173 (1.5%)	1.49 (1.22, 1.81)	<0.001
Thrombosis	339 (3.1%)	301 (2.7%)	1.16 (0.99, 1.35)	0.072
Mental Disorders	476 (8.6%)	491 (5.5%)	1.62 (1.42, 1.85)	<0.001
Rheumatoid Arthritis	23 (0.2%)	21 (0.2%)	1.09 (0.6, 1.97)	0.770
Respiratory Symptoms	3444 (26.1%)	2641 (20.0%)	1.41 (1.33, 1.5)	<0.001
Headache	1271 (9.6%)	789 (6.0%)	1.68 (1.53, 1.84)	<0.001
Fatigue	1756 (13.3%)	1351 (10.2%)	1.35 (1.25, 1.45)	<0.001
Bodyache	617 (4.7%)	443 (3.4%)	1.41 (1.25, 1.6)	<0.001
Diarrhea/constipation	2169 (16.4%)	1262 (9.6%)	1.86 (1.73, 2)	<0.001
Cognitive Impairment	140 (1.1%)	98 (0.7%)	1.43 (1.11, 1.86)	0.006
Disturbances to Smell and Taste	74 (0.6%)	52 (0.4%)	1.43 (1, 2.03)	0.049
Fatigue	1298 (9.8%)	973 (7.4%)	1.37 (1.26, 1.49)	<0.001
Chronic cough	1674 (12.7%)	1088 (8.2%)	1.62 (1.49, 1.75)	<0.001
Brain Fog	326 (2.5%)	209 (1.6%)	1.57 (1.32, 1.88)	<0.001
Palpitations	415 (3.1%)	454 (3.4%)	0.91 (0.8, 1.04)	0.179
Chest pain	2228 (16.9%)	1668 (12.6%)	1.4 (1.31, 1.5)	<0.001
Sexual desire or capacity	35 (0.3%)	22 (0.2%)	1.59 (0.93, 2.72)	0.085
Dizziness	922 (7.0%)	693 (5.2%)	1.36 (1.22, 1.5)	<0.001
Gastrointestinal	3851 (29.1%)	2656 (20.1%)	1.64 (1.54, 1.73)	<0.001
Hair loss	150 (1.1%)	125 (0.9%)	1.2 (0.95, 1.53)	0.130
Abnormal movements	327 (2.5%)	271 (2.1%)	1.21 (1.03, 1.43)	0.021

### 3.3 PASC outcomes in patients with HIV diagnosis by SARS-CoV-2 variant

As shown in **Table 3**, mortality was significantly higher in early pandemic (pre-Delta) period compared with later periods (OR 2.39, 95% CI 1.88-3.05; p < 0.001). The odds of new-onset PASC conditions was also significantly higher in the pre-Delta period: HTN 2.01 (95% CI 1.58-2.57; p < 0.001), DM 2.54 (95% CI 1.90-3.40; p < 0.001), thyroid disorders 2.29 (95% CI 1.39- 3.79; p < 0.001), heart disease 1.98 (95% CI 1.57-2.49; p < 0.001), malignancy 2.46 (95% CI 1.75- 3.45; p < 0.001), thrombosis 1.71 (95% CI 1.29-2.25; p < 0.001), and mental disorders 1.55 (95% CI 1.23-1.96; p < 0.001). There were also significant increases in the prevalence of symptoms among PWH in the pre-Delta compared to later periods: disturbances to smell and taste 3.02 (95% CI 1.61-5.66, p < 0.001), fatigue 1.62, 95% CI 1.4-1.88, p < 0.001), brain fog 1.60 (95% CI 1.2-2.15, p=0.001), palpitations 1.78 (95% CI: 1.38-2.3, p < 0.001), chest pain 1.47 (95% CI: 1.31-1.65, p <0.001), dizziness 1.49 (95% CI: 1.25-1.76, p < 0.001), gastrointestinal symptoms 1.49 (95% CI 1.36-1.64, p <0.001), and abnormal movements 1.61 (95% CI 1.21-2.14, p=0.001).

**Table 3:** PASC outcomes between pre- and since Delta variant COVID-19 patients with HIV positive diagnosis after propensity score matching.

Outcome	Pre-Delta	Since-Delta	OR (95% CI)	p-Value
Mortality	225 (5.2%)	97 (2.2%)	2.39 (1.88, 3.05)	<0.001
Hypertension	203 (9.9%)	105 (5.2%)	2.01 (1.58, 2.57)	<0.001
Diabetes	161 (5.0%)	66 (2.0%)	2.54 (1.9, 3.4)	<0.001
Thyroid Disorders	50 (1.2%)	22 (0.5%)	2.29 (1.39, 3.79)	0.001
Heart Disease	226 (7.6%)	115 (4.0%)	1.98 (1.57, 2.49)	<0.001
Malignancy	115 (3.1%)	48 (1.3%)	2.46 (1.75, 3.45)	<0.001
Thrombosis	139 (3.8%)	82 (2.3%)	1.71 (1.29, 2.25)	<0.001
Mental Disorders	211 (10.2%)	120 (6.8%)	1.55 (1.23, 1.96)	<0.001
Rheumatoid Arthritis	11 (0.3%)	10 (0.2%)*	1.1 (0.47, 2.58)	0.834
Respiratory Symptoms	1249 (28.9%)	898 (20.8%)	1.55 (1.4, 1.71)	<0.001
Headache	466 (10.8%)	322 (7.5%)	1.5 (1.29, 1.74)	<0.001
Bodyache	236 (5.5%)	153 (3.5%)	1.57 (1.28, 1.94)	<0.001
Cognitive Impairment	59 (1.4%)	30 (0.7%)	1.98 (1.27, 3.08)	0.002
Hair loss	61 (1.4%)	31 (0.7%)	1.98 (1.28, 3.06)	0.002
Disturbances to Smell and Taste	39 (0.9%)	13 (0.3%)	3.02 (1.61, 5.66)	<0.001
Fatigue	491 (11.4%)	316 (7.3%)	1.62 (1.4, 1.88)	<0.001
Chronic cough	540 (12.5%)	447 (10.3%)	1.24 (1.08, 1.41)	0.002
Brain Fog	119 (2.8%)	75 (1.7%)	1.6 (1.2, 2.15)	0.001
Palpitations	166 (3.8%)	95 (2.2%)	1.78 (1.38, 2.3)	<0.001
Chest pain	830 (19.2%)	600 (13.9%)	1.47 (1.31, 1.65)	<0.001
Sexual desire or capacity	16 (0.4%)	10 (0.2%)*	1.6 (0.73, 3.53)	0.239
Dizziness	343 (7.9%)	237 (5.5%)	1.49 (1.25, 1.76)	<0.001
Gastrointestinal symptoms	1404 (32.5%)	1053 (24.4%)	1.49 (1.36, 1.64)	<0.001
Abnormal movements	124 (2.9%)	78 (1.8%)	1.61 (1.21, 2.14)	0.001

*TriNetX automatically round patient counts of 1-9, up to 10.

### 3.4 PASC outcomes in vaccinated and unvaccinated patients with HIV diagnosis

The risk of PASC was compared between vaccinated and unvaccinated cohorts of PWH who had a prior diagnosis of COVID-19 (**Table 4**). Notably, the odds for mortality was 0.29 (95% CI 0.19-0.46, p <0.001), diabetes 0.65 (95% CI 0.43-0.99; p = 0.042), heart disease 0.58 (95% CI 0.4-0.85; p = 0.005), and mental disorders 0.66 (95% CI 0.43-1; p = 0.047). Similarly, the odds of persistent symptoms were significantly lower in the vaccinated PWH cohort versus unvaccinated PWH, as follows: respiratory symptoms 0.82 (95% CI 0.7-0.95; p = 0.008), gastrointestinal symptoms 0.78 (95% CI 0.67-0.90; p = 0.001), fatigue (OR: 0.81, 95% CI: 0.67-0.98, p = 0.033), chest pain 0.73 (95% CI 0.61-0.87; p = 0.001) and disturbances of smell and taste 0.47 (95% CI 0.22-1.01; p = 0.047).

**Table 4:** PASC related outcomes between vaccinated and unvaccinated COVID-19 patients with HIV positive diagnosis after propensity score matching.

Outcome	Vaccinated	Unvaccinated	OR (95% CI)	p
Mortality	25 (1.5%)	82 (4.8%)	0.29 (0.19, 0.46)	<0.001
Hypertension	38 (5.8%)	53 (8.2%)	0.68 (0.44, 1.05)	0.081
Diabetes	39 (3.2%)	60 (4.8%)	0.65 (0.43, 0.99)	0.042
Thyroid Disorders	17 (1.1%)	25 (1.6%)	0.68 (0.37, 1.27)	0.223
Heart Disease	46 (4.6%)	79 (7.7%)	0.58 (0.4, 0.85)	0.005
Malignancy	35 (2.6%)	40 (3.1%)	0.85 (0.54, 1.35)	0.492
Thrombosis	36 (2.6%)	44 (3.2%)	0.81 (0.52, 1.27)	0.364
Mental Disorders	37 (7.0%)	67 (10.4%)	0.66 (0.43, 1.00)	0.047
Rheumatoid Arthritis	10 (0.6%)*	10 (0.6%)*	1.00 (0.41, 2.41)	0.998
Respiratory Symptoms	438 (25.5%)	508 (29.6%)	0.82 (0.7, 0.95)	0.008
Headache	156 (9.1%)	188 (10.9%)	0.81 (0.65, 1.02)	0.069
Fatigue	226 (13.2%)	270 (15.7%)	0.81 (0.67, 0.98)	0.033
Bodyache	76 (4.4%)	81 (4.7%)	0.94 (0.68, 1.29)	0.683
Cognitive Impairment	18 (1.0%)	22 (1.3%)	0.82 (0.44, 1.53)	0.525
Disturbances to Smell and Taste	10 (0.6%)	21 (1.2%)	0.47 (0.22, 1.01)	0.047
Fatigue	174 (10.1%)	195 (11.4%)	0.88 (0.71, 1.09)	0.247
Chronic cough	221 (12.9%)	247 (14.4%)	0.88 (0.72, 1.07)	0.196
Brain Fog	49 (2.9%)	50 (2.9%)	0.98 (0.66, 1.46)	0.919
Palpitations	54 (3.1%)	60 (3.5%)	0.9 (0.62, 1.3)	0.568
Chest pain	250 (14.6%)	326 (19.0%)	0.73 (0.61, 0.87)	0.001
Sexual desire or capacity	10 (0.6%)*	10 (0.6%)*	1.00 (0.42, 2.41)	1.000
Dizziness	121 (7.0%)	129 (7.5%)	0.93 (0.72, 1.21)	0.599
Gastrointestinal	480 (27.9%)	572 (33.3%)	0.78 (0.67, 0.9)	0.001
Hair loss	22 (1.3%)	21 (1.2%)	1.05 (0.57, 1.91)	0.878
Abnormal movements	50 (2.9%)	56 (3.3%)	0.89 (0.6, 1.31)	0.554

*TriNetX automatically round patient counts of 1-9, up to 10.

## 4 Discussion

In this study from a large health network in the United States, we showed that compared to the general population, HIV infection was significantly associated with increased odds of experiencing PASC, as defined by either persistent COVID-attributable symptoms or developing new conditions at least 28 days after the initial COVID-19 diagnosis. We further showed COVID-19 vaccination had a protective effect against the development of new-onset conditions among PWH. Studies describing the association between HIV status and the emergence of PASC conditions or the impact of COVID-19 vaccination on PASC severity are limited. In an earlier study, we previously showed that vaccination significantly lowered the odds of PASC in vaccinated COVID-19 survivors in the general population (25), however, to the best of our knowledge, this is the first study to demonstrate the protective effect of COVID-19 vaccination against PASC among PWH. This is a crucial finding, as the management of PASC currently remains quite challenging and fragmented. With the approach to managing PASC likely to evolve with the accumulation of new evidence, our findings highlight the role of vaccination as an effective preventive approach to address a burgeoning public health problem.

The results of our study further showed that PASC manifestations were common among COVID-19 survivors even after propensity score matching and encompassed a wide range of heterogenous and overlapping clinical findings in multiple organ systems including hematolo-oncological disorders (malignancies and thrombosis), cardiovascular disorders (HTN and heart disease), respiratory symptoms, gastrointestinal disorders, neurocognitive impairment and mental health disorders. Among PWH, the odds of developing new medical conditions was lowest for HTN (1.18-fold increase) and highest for gastrointestinal symptoms (1.64-fold increase) and constitutional signs/symptoms related to general wellbeing such as headaches (1.68-fold increase). Similarly, the odds of new-onset heart disease and malignancy among PWH was similarly elevated significantly. However, contrary to other studies, there was no increased odds of developing DM, thyroid diseases or rheumatological disorders. Nonetheless, overall, these findings are consistent with findings from multiple studies, which have reported one or more new or persistent physical and/or mental health conditions in 6% to 90% of COVID-19 survivors up to one year after the initial acute infection (4–9, 28, 29).

The increased odds of malignancies among PWH after SARS-CoV-2 infection is an interesting finding that warrants further discussion. HIV is a well-recognized risk factor for both AIDS-defining and non-AIDS defining cancers despite successful treatment (30). However, although common molecular signaling pathways between SARS-CoV-2 and cancer have been noted (31), a clear association has not been established between the two entities. We therefore hypothesize that any potential role SARS-CoV-2 may play in increasing cancer risk in PWH may be a contributory rather than a primary effect, by amplifying the well-described mechanisms and pathways through which HIV is known to elevate the risk of both AIDS-defining and non-AIDS defining cancers (32). More research is needed to investigate this assertion.

The pathophysiologic mechanisms underlying PASC are poorly understood, however, current evidence suggests a prolonged systemic inflammation and an aberrant immune response as major contributing factors (2, 33, 34). Furthermore, in PWH specifically, it may be difficult to distinguish between the relative contributions of HIV-specific factors (i.e., HIV-associated gut dysfunction, immune activation, proinflammatory state and ART-related toxicities) (17, 18) from the effects of the intense systemic inflammatory response triggered by acute SARS-CoV-2 infection (34). Nonetheless, it is hypothesized that the binding of SARS-CoV-2 to the angiotensin-converting enzyme 2 (ACE2) receptor results in activation of the renin-angiotensin pathway which has been implicated in new-onset HTN, acute myocardial injury, cardiac arrhythmias and acute coronary artery events (2, 35). Other studies have suggested a more direct pathophysiologic mechanism through SARS-CoV-2-induced injury to cardiomyocytes, pneumocytes, endothelial cells, pancreatic islet cells and neurons (34, 35). This may lead to fibrosis, structural remodeling and prolonged end-organ dysfunction and unfavorable outcomes associated with PASC manifestations (34, 35). Despite these assertions, it remains unclear whether PASC manifestations are entirely attributable to the direct effects of SARS-CoV-2 infection, or whether infection with the virus results in unmasking or decompensation of pre-existing subclinical health conditions. It also remains unclear whether new-onset conditions that arise from SARS-CoV-2 infection are permanent or resolve over time.

Our findings further suggested that the odds of PASC may depend on the type of SARS-CoV-2 variants circulating in the population. Among PWH, infection with the wild-type variants earlier on in the pre-Delta phases of the pandemic was associated with 3-fold higher odds of mortality and a 2- to 3-fold higher odds of new-onset PASC conditions or persistent symptoms, compared with the Delta or Omicron variant pandemic period. These findings corroborate recent reports from large observational studies from the United Kingdom and Italy (13, 14). Several reasons could explain these observations. For example, while new mutations in Omicron sublineages confer greater replicative advantage (i.e., increased infectiousness), several studies have reported milder disease compared with Delta and prior variants, which could partly account for lower odds of PASC (36, 37). Another plausible explanation why PASC occurrence rates were lower with each successive wave of SARS-CoV-2 variant could be attributed in part to the protective effect of COVID-19 vaccination as it became more widely available for the population. More detailed studies are needed to confirm these findings.

Our study had a few methodological limitations worth discussing, which may affect the generalizability of our findings. First, people with asymptomatic or mild disease are less likely to seek medical care and therefore unlikely to have been captured in the database, leading to an underestimate of the true prevalence of PASC among COVID-19 survivors. Second, there are varying definitions of PASC and currently no standardized system of reporting symptoms or conditions. In addition, PASC documentation relies heavily on self-reports, which may have limited the accuracy of reporting in the EHR system. Third, we were unable to determine the type of COVID-19 vaccine and number of doses received by patients (i.e., first, second, or booster doses) both of which may have an impact on the occurrence and severity of PASC outcomes. Due to the nature of the database, a patients’ first or second dose may have been missed in the reporting (e.g., if a dose occurred outside a patients’ primary HCO, such as a pharmacy or mass vaccination event, and was not reported back), but included a record of a booster. We addressed this limitation in the EHR system by assuming that these patients have a first/second dose, and thus included them as a “vaccinated” patient. Fourth, we were able to assess onset and severity of symptoms, both which are known to be associated with HIV positive status as well as occurrence and severity of PASC. Fifth, in assessing incident PASC, we compared PWH with those without HIV. HIV positive status is itself is associated with several of the symptoms and comorbidities included in PASC, which may make it challenging to distinguish between complications related to SARS-CoV-2 and the progression of HIV. Another limitation of our study was the inability to evaluate the effect of COVID-19 reinfections on PASC, as reinfections are linked to an increased risk of PASC (38). This limitation stemmed from the lack of ICD-10 codes for distinguishing first COVID-19 infections from reinfections. Finally, being an observational study, causation cannot be inferred. Despite these limitations, however, major strengths of our study lie in the fact that we were able to demonstrate higher odds of PASC among PWH. Moreover, in addition to symptoms, we were able to carefully capture the emergence of new-onset health conditions following acute SARS-CoV-2 infection. Our findings may have implications outside of HIV as other clinical conditions known to be associated with chronic sustained inflammation, such as rheumatoid arthritis and systemic lupus erythematosus, may suffer from higher PASC and should be investigated separately.

In summary, our study showed that PWH have higher odds of PASC compared with their non-HIV counterparts. Importantly, we also demonstrated that prior COVID-19 vaccination was associated with significantly lower odds of all-cause mortality and was protective against the development of PASC among PWH. With the increase in the number of COVID-19 survivors, vaccination may offer an effective preventive strategy to address a burgeoning public health problem.

## Data availability statement

The original contributions presented in the study are included in the article/**Supplementary Material**. Further inquiries can be directed to the corresponding author.

## Ethics statement

The studies involving humans were approved by University Hospitals Cleveland Medical Center Institutional Review Board and the studies were conducted in accordance with the local legislation and institutional requirements. The ethics committee/institutional review board waived the requirement of written informed consent for participation from the participants or the participants’ legal guardians/next of kin because The TritNetX Research Network was used to obtained de-identified data.

## Author contributions

GY: Conceptualization, Formal Analysis, Investigation, Methodology, Validation, Writing – original draft, Writing – review & editing, Project administration, Visualization, Data curation. JP: Conceptualization, Data curation, Formal Analysis, Investigation, Methodology, Project administration, Validation, Writing – original draft, Writing – review & editing. NP: Conceptualization, Data curation, Formal Analysis, Investigation, Methodology, Software, Writing – original draft, Writing – review & editing. GM: Conceptualization, Data curation, Formal Analysis, Funding acquisition, Investigation, Methodology, Project administration, Resources, Software, Supervision, Validation, Visualization, Writing – original draft, Writing – review & editing.
