# Supplementary material for: Associations between post-acute sequelae of SARS-CoV-2, COVID-19 vaccination and HIV infection: a United States cohort study
Source: Front Immunol. 2024 Jan 22;15:1297195. doi: 10.3389/fimmu.2024.1297195 (PMC10838972; doi:10.3389/fimmu.2024.1297195)
Supplement: Supplementary file 1 [file DataSheet_1.docx]

Supplementary Material

Supplemental Table 1. Cohort inclusion and exclusion criteria, including code and code system

| Characteristic | Coding system | Code(s) |
| --- | --- | --- |
| HIV | ICD-10 | B20 |
| HIV 1 RNA viral load (≥ 1000 copies/ml) | LOINC | 70241-5, 20447-9, 32515-9, 25836-8, 41513-3, 21008-8, 59419-2, 48551-6, 48511-0, 41498-7 |
| SARS coronavirus 2 and related RNA [Positive Presence] | LOINC | 94307-6, 94308-4, 94039-2, 94310-0, 94314-2, 94315-9, 94316-7, 94500-6, 94502-2, 94533-7, 94534-5, 94559-2, 94565-9, 94639-2, 94647-5, 94758-0, 94759-8, 94760-6, 94845-5, 95406-5, 95409-9, 95608-6, 96123-5, 96763-8, 94558-4, 95209-3, 96119-3, 94763-0, 97097-0 |
| COVID-19 Diagnosis | ICD-10 | U07.1, U07.2, J12.82 |
| SARS coronavirus 2 vaccine | LOINC | 91300, 91301, 91302, 91303, 91305, 91306, 91307, 91309, 91316, 91317, 91315, 91314, 91313, 91312, 91311, 91301 |
|  | ICD-10-PCS | XW023W7, XW023V7, XW023U6, XW023T6, XW023S6, XW013W7, XW013U6, XW013V7, XW013T6, XW013S6 |
|  | RxNorm | 2610328, 2610319, 2610347, 2623378, 2623382, 2606074, 2479831 |

Supplemental Table 2. Baseline characteristics and outcomes

| Baseline characteristics | Coding system | Code(s) |
| --- | --- | --- |
| Diabetes mellitus | ICD-10 | E08-E13 |
| Chronic obstructive pulmonary disease | ICD-10 | J44 |
| Asthma | ICD-10 | J45 |
| Coronary artery disease | ICD-10 | I25.10 |
| Human immunodeficiency virus | ICD-10 | B20 |
| Neoplasm | ICD-10 | C00-D49 |
| Transplanted organ and tissue status | ICD-10 | Z94 |
| Chronic kidney disease | ICD-10 | N18 |
| BMI | LOINC | 39156-5 |
| Outcomes | | |
| Hypertensive diseases | ICD-10 | I10-I16 |
| Diabetes mellitus | ICD-10 | E08-E13 |
| Thyroid disease | ICD-10 | E06, E03 |
| Heart disease | ICD-10 | I30-I52, I21 |
| Malignant neoplasms | ICD-10 | C00-C14, C15-C26, C30-C39, C40-C41, C43-C44, C45-C49, C50, C51-C58, C60-C63, C64-C68, C69-C72, C73-C75, C76-C80, C7A, C7B, C81-C96 |
| Thrombosis | ICD-10 | I82, I60-I69 |
| Mental Disorders | ICD-10 | F40-F48, F30-F39 |
| Rheumatoid arthritis | ICD-10 | M06.9 |
| Respiratory symptoms | ICD-10 | R06, R05 |
| Headache | ICD-10 | R51 |
| Fatigue | ICD-10 | R53.1, R53.81, R53.83 |
| Body ache | ICD-10 | R52 |
| Diarrhea/constipation | ICD-10 | R19.7, K59.0 |
| Neurocognitive disfunction | ICD-10 | R41.840, G31.84 |
| Disturbances to smell and taste | ICD-10 | R43 |
| Chronic cough | ICD-10 | R05.3, R05.4, R05.8, R05.9 |
| Brain Fog | ICD-10 | R41.89, R41.9, R41.840, G31.84 |
| Palpitations | ICD-10 | R00.2 |
| Chest pain | ICD-10 | R07.1, R07.2, R07.8, R07.9 |
| Sexual desire | ICD-10 | F52 |
| Dizziness | ICD-10 | R42 |
| Gastrointestinal | ICD-10 | R10, R11, R12, R13, R14, R15, R19 |
| Hair loss | ICD-10 | L65 |
| Abnormal movements | ICD-10 | R25.0, R25.1, R25.2, R25.3, R25.8, R25.9 |

Supplemental Table 3. Patient counts for HIV medication use within COVID-19 and HIV+ cohort, before and after propensity score matching.

| HIV Medication  N = 10946 (82.8%) | HIV + (Before Matching)  N = 13214 | HIV + (After Matching)  N = 13212 |
| --- | --- | --- |
| emtricitabine | 7959 (60.2%) | 7957 (60.2%) |
| Integrase inhibitors | 6422 (48.6%) | 6420 (48.6%) |
| tenofovir alafenamide | 5965 (45.1%) | 5964 (45.1%) |
| tenofovir disoproxil | 5675 (42.9%) | 5673 (42.9%) |
| dolutegravir | 4360 (33.0%) | 4358 (33.0%) |
| Protease inhibitors | 3601 (27.3%) | 3600 (27.2%) |
| lamivudine | 3309 (25.0%) | 3307 (25.0%) |
| atorvastatin | 3175 (24.0%) | 3173 (24.0%) |
| bictegravir | 3139 (23.8%) | 3139 (23.8%) |
| ritonavir | 2919 (22.1%) | 2918 (22.1%) |
| cobicistat | 2892 (21.9%) | 2892 (21.9%) |
| abacavir | 2618 (19.8%) | 2616 (19.8%) |
| darunavir | 2047 (15.5%) | 2047 (15.5%) |
| elvitegravir | 2002 (15.2%) | 2002 (15.2%) |
| efavirenz | 1894 (14.3%) | 1894 (14.3%) |
| tenofovir | 1755 (13.3%) | 1755 (13.3%) |
| rilpivirine | 1453 (11.0%) | 1451 (11.0%) |
| raltegravir | 1411 (10.7%) | 1410 (10.7%) |
| atazanavir | 1182 (8.9%) | 1181 (8.9%) |
| rosuvastatin | 1142 (8.6%) | 1141 (8.6%) |
| pravastatin | 1032 (7.8%) | 1030 (7.8%) |
| zidovudine | 790 (6.0%) | 790 (6.0%) |
| lopinavir | 488 (3.7%) | 488 (3.7%) |
| simvastatin | 487 (3.7%) | 487 (3.7%) |
| fosamprenavir | 129 (1.0%) | 129 (1.0%) |
| cabotegravir | 69 (0.5%) | 69 (0.5%) |
| pitavastatin | 53 (0.4%) | 53 (0.4%) |
| indinavir | 52 (0.4%) | 52 (0.4%) |
| lovastatin | 49 (0.4%) | 49 (0.4%) |
| saquinavir | 41 (0.3%) | 41 (0.3%) |
| tipranavir | 11 (0.1%) | 11 (0.1%) |
| fluvastatin | 10 (0.1%)* | 10 (0.1%)* |
| * TriNetX automatically round patient counts of 1-9, up to 10 | | |

Supplemental table 4. PASC related outcomes between COVID-19 patients with or without a HIV positive diagnosis (HIV+, HIV-), before propensity score matching.

| Outcome | HIV + | HIV - | OR (95% CI) | p-Value |
| --- | --- | --- | --- | --- |
| Mortality | 447 (3.3%) | 47730 (1.5%) | 2.21 (2.01, 2.43) | <0.001 |
| Hypertension | 460 (7.3%) | 95094 (4.4%) | 1.71 (1.55, 1.88) | <0.001 |
| Diabetes | 336 (3.3%) | 49508 (1.9%) | 1.81 (1.63, 2.02) | <0.001 |
| Thyroid Disorders | 118 (1.0%) | 32099 (1.1%) | 0.84 (0.7, 1.01) | 0.065 |
| Heart Disease | 532 (6.1%) | 87922 (3.5%) | 1.76 (1.61, 1.92) | <0.001 |
| Malignancy | 251 (2.2%) | 35774 (1.3%) | 1.8 (1.59, 2.04) | <0.001 |
| Thrombosis | 340 (3.0%) | 44939 (1.6%) | 1.95 (1.75, 2.17) | <0.001 |
| Mental Disorders | 480 (8.5%) | 115191 (5.1%) | 1.74 (1.59, 1.91) | <0.001 |
| Rheumatoid Arthritis | 23 (0.2%) | 4968 (0.2%) | 1.07 (0.71, 1.62) | 0.735 |
| Respiratory Symptoms | 3487 (26.1%) | 438066 (14.2%) | 2.14 (2.06, 2.22) | <0.001 |
| Headache | 1277 (9.5%) | 143557 (4.6%) | 2.17 (2.05, 2.3) | <0.001 |
| Fatigue | 1775 (13.3%) | 234214 (7.6%) | 1.87 (1.78, 1.97) | <0.001 |
| Bodyache | 622 (4.7%) | 67698 (2.2%) | 2.18 (2.01, 2.37) | <0.001 |
| Diarrhea/constipation | 2192 (16.4%) | 197046 (6.4%) | 2.88 (2.75, 3.02) | <0.001 |
| Cognitive Impairment | 142 (1.1%) | 18529 (0.6%) | 1.78 (1.51, 2.1) | <0.001 |
| Disturbances to Smell and Taste | 74 (0.6%) | 11196 (0.4%) | 1.53 (1.22, 1.93) | <0.001 |
| Fatigue | 1310 (9.8%) | 187080 (6.0%) | 1.69 (1.59, 1.79) | <0.001 |
| Chronic cough | 1692 (12.7%) | 191805 (6.2%) | 2.19 (2.08, 2.31) | <0.001 |
| Brain Fog | 328 (2.5%) | 37487 (1.2%) | 2.05 (1.84, 2.29) | <0.001 |
| Palpitations | 417 (3.1%) | 84840 (2.7%) | 1.14 (1.04, 1.26) | 0.008 |
| Chest pain | 2243 (16.8%) | 241491 (7.8%) | 2.38 (2.28, 2.49) | <0.001 |
| Sexual desire or capacity | 36 (0.3%) | 2919 (0.1%) | 2.86 (2.06, 3.97) | <0.001 |
| Dizziness | 934 (7.0%) | 123103 (4.0%) | 1.81 (1.7, 1.94) | <0.001 |
| Gastrointestinal | 3892 (29.1%) | 483134 (15.6%) | 2.22 (2.14, 2.3) | <0.001 |
| Hair loss | 151 (1.1%) | 25048 (0.8%) | 1.4 (1.19, 1.64) | <0.001 |
| Abnormal movements | 332 (2.5%) | 40492 (1.3%) | 1.92 (1.72, 2.14) | <0.001 |

Supplemental table 5. Baseline characteristics between Pre- and Since Delta variant COVID-19 patients with HIV positive diagnosis, both before and after propensity score matching.

|  | Before Matching | | | After Matching | | |
| --- | --- | --- | --- | --- | --- | --- |
| Characteristic Name | Pre-Delta | Since-Delta | p-Value | Pre-Delta | Since-Delta | p-Value |
| N | 4341 | 8371 |  | 4321 | 4321 |  |
| Age | 49.16 ± 13.66 | 49.43 ± 13.82 | 0.288 | 49.14 ± 13.68 | 48.98 ± 13.61 | 0.592 |
| At least 50 years | 2288 (52.7%) | 4385 (52.4%) | 0.729 | 2271 (52.6%) | 2249 (52.0%) | 0.636 |
| At least 65 years | 552 (12.7%) | 1178 (14.1%) | 0.034 | 552 (12.8%) | 539 (12.5%) | 0.674 |
| Male | 2947 (67.9%) | 5735 (68.5%) | 0.474 | 2930 (67.8%) | 2962 (68.5%) | 0.460 |
| Not Hispanic or Latino | 2800 (64.5%) | 5663 (67.7%) | <0.001 | 2800 (64.8%) | 2799 (64.8%) | 0.982 |
| Black or African American | 2017 (46.5%) | 3509 (41.9%) | <0.001 | 2016 (46.7%) | 2020 (46.7%) | 0.931 |
| Female | 1394 (32.1%) | 2634 (31.5%) | 0.457 | 1391 (32.2%) | 1359 (31.5%) | 0.460 |
| White | 1384 (31.9%) | 3235 (38.6%) | <0.001 | 1380 (31.9%) | 1396 (32.3%) | 0.712 |
| Unknown Ethnicity | 895 (20.6%) | 1866 (22.3%) | 0.030 | 895 (20.7%) | 901 (20.9%) | 0.874 |
| Unknown Race | 871 (20.1%) | 1483 (17.7%) | 0.001 | 856 (19.8%) | 844 (19.5%) | 0.745 |
| Hispanic or Latino | 646 (14.9%) | 842 (10.1%) | <0.001 | 626 (14.5%) | 621 (14.4%) | 0.878 |
| Asian | 44 (1.0%) | 92 (1.1%) | 0.657 | 44 (1.0%) | 40 (0.9%) | 0.661 |
| American Indian or  Alaska Native | 20 (0.5%) | 42 (0.5%) | 0.753 | 20 (0.5%) | 18 (0.4%) | 0.745 |
| Native Hawaiian or  Other Pacific Islander | 10 (0.2%) | 10 (0.1%) | 0.135 | 10 (0.2%) | 10 (0.2%) | 1.000 |
| Unknown Gender | 0 (0.0%) | 10 (0.1%) | 0.023 | 0 (0.0%) | 0 (0.0%) |  |
| Hypertensive diseases | 2249 (51.8%) | 4339 (51.8%) | 0.978 | 2240 (51.8%) | 2259 (52.3%) | 0.682 |
| Neoplasms | 1470 (33.9%) | 3285 (39.2%) | <0.001 | 1470 (34.0%) | 1479 (34.2%) | 0.838 |
| Hyperlipidemia | 1397 (32.2%) | 2895 (34.6%) | 0.007 | 1396 (32.3%) | 1373 (31.8%) | 0.596 |
| Overweight and obesity | 1240 (28.6%) | 2247 (26.8%) | 0.039 | 1230 (28.5%) | 1238 (28.7%) | 0.849 |
| Diabetes mellitus | 1091 (25.1%) | 1931 (23.1%) | 0.010 | 1084 (25.1%) | 1067 (24.7%) | 0.672 |
| Chronic kidney disease | 759 (17.5%) | 1654 (19.8%) | 0.002 | 758 (17.5%) | 742 (17.2%) | 0.650 |
| Ischemic heart diseases | 696 (16.0%) | 1434 (17.1%) | 0.116 | 695 (16.1%) | 635 (14.7%) | 0.074 |
| COPD | 442 (10.2%) | 962 (11.5%) | 0.025 | 442 (10.2%) | 409 (9.5%) | 0.233 |
| Organ Transplant History | 114 (2.6%) | 307 (3.7%) | 0.002 | 113 (2.6%) | 110 (2.5%) | 0.839 |
| CD3+CD4+ (T4 helper),  LOINC: 24467-3 | 597.98 ± 365.64 | 576.72 ± 393.24 | 0.032 | 598.53 ± 365.87 | 585.66 ± 392.27 | 0.253 |
| At most 200 /uL | 704 (31.2%) | 1589 (35.7%) | <0.001 | 698 (31.1%) | 783 (34.1%) | 0.015 |
| BMI | 28.98 ± 6.6 | 28.32 ± 6.76 | 0.002 | 28.98 ± 6.61 | 28.56 ± 6.87 | 0.084 |
| At least 25 kg/m2 | 1279 (29.5%) | 2625 (31.4%) | 0.028 | 1273 (29.5%) | 1248 (28.9%) | 0.554 |
| At least 30 kg/m2 | 821 (18.9%) | 1592 (19.0%) | 0.886 | 815 (18.9%) | 812 (18.8%) | 0.934 |
| HIV 1 RNA (viral load),  LOINC: 20447-9 | 18267.53 ± 91079.24 | 21787.85 ± 96627.89 | 0.334 | 18320.18 ± 91206.55 | 22347.07 ± 98332.47 | 0.337 |
| At most 50 {copies}/mL | 670 (64.8%) | 1216 (62.0%) | 0.172 | 669 (64.9%) | 628 (61.9%) | 0.217 |
| HIV 1 RNA (viral load),  LOINC: 70241-5 | 13830.09 ± 69972.56 | 18056.82 ± 103502.41 | 0.481 | 13987.92 ± 70359.09 | 24562.32 ± 128539.77 | 0.167 |
| At most 50 {copies}/mL | 283 (80.4%) | 683 (76.7%) | 0.001 | 280 (80.5%) | 330 (73.0%) | 0.036 |
| COVID 19 Vaccine | 30 (0.7%) | 989 (11.8%) | <0.001 | 30 (0.7%) | 513 (11.9%) | <0.001 |

Supplemental Table 6. Patient counts for HIV medication between Pre and Since Delta COVID-19 and HIV+ cohorts, before and after propensity score matching.

| HIV Medication  Pre-Delta: N = 3521 (81.1%)  Since-Delta: N = 6984 (83.4%) | Before Matching | | | After Matching | | |
| --- | --- | --- | --- | --- | --- | --- |
| Characteristic Name | Pre-Delta | Since-Delta | p-Value | Pre-Delta | Since-Delta | p-Value |
| N | 4341 | 8371 |  | 4321 | 4321 |  |
| emtricitabine | 2555 (58.9%) | 5085 (60.7%) | 0.039 | 2543 (58.9%) | 2635 (61.0%) | 0.043 |
| Integrase inhibitors | 2127 (49.0%) | 4014 (48.0%) | 0.263 | 2117 (49.0%) | 2013 (46.6%) | 0.025 |
| tenofovir alafenamide | 1884 (43.4%) | 3837 (45.8%) | 0.009 | 1873 (43.3%) | 1984 (45.9%) | 0.016 |
| tenofovir disoproxil | 1855 (42.7%) | 3580 (42.8%) | 0.970 | 1847 (42.7%) | 1839 (42.6%) | 0.862 |
| dolutegravir | 1365 (31.4%) | 2796 (33.4%) | 0.026 | 1357 (31.4%) | 1395 (32.3%) | 0.380 |
| Protease inhibitors | 1078 (24.8%) | 2344 (28.0%) | <0.001 | 1071 (24.8%) | 1200 (27.8%) | 0.002 |
| lamivudine | 1032 (23.8%) | 2131 (25.5%) | 0.037 | 1029 (23.8%) | 1043 (24.1%) | 0.724 |
| cobicistat | 983 (22.6%) | 1801 (21.5%) | 0.144 | 977 (22.6%) | 912 (21.1%) | 0.091 |
| atorvastatin | 975 (22.5%) | 2044 (24.4%) | 0.014 | 975 (22.6%) | 1015 (23.5%) | 0.307 |
| bictegravir | 907 (20.9%) | 2100 (25.1%) | <0.001 | 901 (20.9%) | 1105 (25.6%) | <0.001 |
| ritonavir | 849 (19.6%) | 1919 (22.9%) | <0.001 | 845 (19.6%) | 982 (22.7%) | <0.001 |
| abacavir | 839 (19.3%) | 1658 (19.8%) | 0.519 | 835 (19.3%) | 794 (18.4%) | 0.259 |
| elvitegravir | 713 (16.4%) | 1212 (14.5%) | 0.004 | 710 (16.4%) | 613 (14.2%) | 0.004 |
| efavirenz | 664 (15.3%) | 1135 (13.6%) | 0.008 | 662 (15.3%) | 579 (13.4%) | 0.011 |
| darunavir | 654 (15.1%) | 1296 (15.5%) | 0.537 | 649 (15.0%) | 656 (15.2%) | 0.833 |
| tenofovir | 569 (13.1%) | 1146 (13.7%) | 0.362 | 564 (13.1%) | 607 (14.0%) | 0.177 |
| raltegravir | 456 (10.5%) | 865 (10.3%) | 0.764 | 456 (10.6%) | 405 (9.4%) | 0.067 |
| rilpivirine | 424 (9.8%) | 973 (11.6%) | 0.002 | 424 (9.8%) | 503 (11.6%) | 0.006 |
| atazanavir | 394 (9.1%) | 713 (8.5%) | 0.289 | 393 (9.1%) | 372 (8.6%) | 0.426 |
| pravastatin | 342 (7.9%) | 636 (7.6%) | 0.573 | 342 (7.9%) | 318 (7.4%) | 0.331 |
| rosuvastatin | 333 (7.7%) | 751 (9.0%) | 0.013 | 332 (7.7%) | 359 (8.3%) | 0.284 |
| zidovudine | 243 (5.6%) | 503 (6.0%) | 0.350 | 243 (5.6%) | 244 (5.6%) | 0.963 |
| simvastatin | 163 (3.8%) | 295 (3.5%) | 0.508 | 163 (3.8%) | 144 (3.3%) | 0.270 |
| lopinavir | 152 (3.5%) | 297 (3.5%) | 0.893 | 152 (3.5%) | 146 (3.4%) | 0.724 |
| fosamprenavir | 48 (1.1%) | 76 (0.9%) | 0.282 | 47 (1.1%) | 36 (0.8%) | 0.225 |
| lovastatin | 19 (0.4%) | 29 (0.3%) | 0.426 | 19 (0.4%) | 12 (0.3%) | 0.208 |
| pitavastatin | 17 (0.4%) | 33 (0.4%) | 0.982 | 17 (0.4%) | 14 (0.3%) | 0.589 |
| saquinavir | 15 (0.3%) | 21 (0.3%) | 0.341 | 15 (0.3%) | 10 (0.2%) | 0.317 |
| indinavir | 14 (0.3%) | 33 (0.4%) | 0.528 | 14 (0.3%) | 11 (0.3%) | 0.548 |
| fluvastatin | 10 (0.2%) | 10 (0.1%) | 0.135 | 10 (0.2%) | 10 (0.2%) | 1.000 |
| tipranavir | 10 (0.2%) | 10 (0.1%) | 0.135 | 10 (0.2%) | 10 (0.2%) | 1.000 |
| cabotegravir | 0 (0.0%) | 72 (0.9%) | <0.001 | 0 (0.0%) | 37 (0.9%) | <0.001 |

Supplemental table 7. PASC related outcomes between Pre and Since Delta COVID-19 and HIV+ cohorts, before propensity score matching.

| Outcome | Pre-Delta | Since-Delta | OR (95% CI) | p-Value |
| --- | --- | --- | --- | --- |
| Mortality | 226 (5.2%) | 194 (2.3%) | 2.34 (1.92, 2.84) | <0.001 |
| Hypertension | 207 (9.9%) | 206 (5.1%) | 2.06 (1.68, 2.51) | <0.001 |
| Diabetes | 162 (5.0%) | 134 (2.1%) | 2.5 (1.98, 3.16) | <0.001 |
| Thyroid Disorders | 50 (1.2%) | 51 (0.7%) | 1.9 (1.28, 2.81) | 0.001 |
| Heart Disease | 228 (7.6%) | 243 (4.4%) | 1.79 (1.49, 2.16) | <0.001 |
| Malignancy | 116 (3.1%) | 102 (1.4%) | 2.18 (1.66, 2.85) | <0.001 |
| Thrombosis | 141 (3.8%) | 158 (2.2%) | 1.74 (1.38, 2.19) | <0.001 |
| Mental Disorders | 213 (10.2%) | 219 (6.4%) | 1.65 (1.36, 2.01) | <0.001 |
| Rheumatoid Arthritis | 11 (0.3%) | 11 (0.1%) | 1.94 (0.84, 4.48) | 0.113 |
| Respiratory Symptoms | 1259 (28.8%) | 1802 (21.2%) | 1.51 (1.39, 1.64) | <0.001 |
| Headache | 469 (10.7%) | 629 (7.4%) | 1.51 (1.33, 1.71) | <0.001 |
| Fatigue | 672 (15.4%) | 876 (10.3%) | 1.58 (1.42, 1.77) | <0.001 |
| Bodyache | 236 (5.4%) | 295 (3.5%) | 1.59 (1.34, 1.9) | <0.001 |
| Diarrhea/constipation | 781 (17.9%) | 1156 (13.6%) | 1.39 (1.25, 1.53) | <0.001 |
| Cognitive Impairment | 59 (1.4%) | 61 (0.7%) | 1.9 (1.32, 2.72) | <0.001 |
| Disturbances to Smell and Taste | 39 (0.9%) | 24 (0.3%) | 3.19 (1.91, 5.3) | <0.001 |
| Fatigue | 494 (11.3%) | 636 (7.5%) | 1.58 (1.4, 1.79) | <0.001 |
| Chronic cough | 546 (12.5%) | 886 (10.4%) | 1.23 (1.1, 1.38) | <0.001 |
| Brain Fog | 119 (2.7%) | 155 (1.8%) | 1.51 (1.19, 1.92) | 0.001 |
| Palpitations | 168 (3.8%) | 199 (2.3%) | 1.67 (1.36, 2.06) | <0.001 |
| Chest pain | 835 (19.1%) | 1140 (13.4%) | 1.53 (1.39, 1.69) | <0.001 |
| Sexual desire or capacity | 16 (0.4%) | 12 (0.1%) | 2.6 (1.23, 5.51) | 0.009 |
| Dizziness | 345 (7.9%) | 469 (5.5%) | 1.47 (1.27, 1.7) | <0.001 |
| Gastrointestinal | 1412 (32.4%) | 2085 (24.5%) | 1.47 (1.36, 1.6) | <0.001 |
| Hair loss | 61 (1.4%) | 68 (0.8%) | 1.76 (1.24, 2.49) | 0.001 |
| Abnormal movements | 124 (2.8%) | 159 (1.9%) | 1.53 (1.21, 1.95) | <0.001 |

Supplemental Table 8. Baseline characteristics between vaccinated and unvaccinated COVID-19 HIV positive cohorts, both before and after propensity score matching.

|  | Before Matching | | | After Matching | | |
| --- | --- | --- | --- | --- | --- | --- |
| Characteristic Name | Vaccinated | Unvaccinated | p-Value | Vaccinated | Unvaccinated | p-Value |
| N | 1732 | 11463 |  | 1718 | 1718 |  |
| Age | 52.23 ± 13.57 | 48.96 ± 13.68 | <0.001 | 52.17 ± 13.53 | 52.16 ± 13.6 | 0.980 |
| At least 50 years | 1060 (61.2%) | 5911 (51.6%) | <0.001 | 1049 (61.1%) | 1062 (61.8%) | 0.649 |
| At least 65 years | 311 (18.0%) | 1460 (12.7%) | <0.001 | 306 (17.8%) | 320 (18.6%) | 0.536 |
| Not Hispanic or Latino | 1385 (80.0%) | 7351 (64.1%) | <0.001 | 1372 (79.9%) | 1384 (80.6%) | 0.607 |
| Male | 1280 (73.9%) | 7697 (67.1%) | <0.001 | 1267 (73.7%) | 1298 (75.6%) | 0.224 |
| White | 824 (47.6%) | 3954 (34.5%) | <0.001 | 818 (47.6%) | 826 (48.1%) | 0.785 |
| Black or African American | 614 (35.5%) | 5117 (44.6%) | <0.001 | 614 (35.7%) | 623 (36.3%) | 0.749 |
| Female | 451 (26.0%) | 3765 (32.8%) | <0.001 | 451 (26.3%) | 419 (24.4%) | 0.209 |
| Unknown Race | 242 (14.0%) | 2220 (19.4%) | <0.001 | 242 (14.1%) | 230 (13.4%) | 0.552 |
| Hispanic or Latino | 241 (13.9%) | 1307 (11.4%) | 0.002 | 240 (14.0%) | 231 (13.4%) | 0.655 |
| Unknown Ethnicity | 106 (6.1%) | 2805 (24.5%) | <0.001 | 106 (6.2%) | 103 (6.0%) | 0.830 |
| Asian | 38 (2.2%) | 108 (0.9%) | <0.001 | 32 (1.9%) | 32 (1.9%) | 1.000 |
| American Indian or Alaska Native | 12 (0.7%) | 50 (0.4%) | 0.145 | 10 (0.6%) | 10 (0.6%) | 1.000 |
| Native Hawaiian or Other Pacific Islander | 10 (0.6%) | 14 (0.1%) | <0.001 | 10 (0.6%) | 0 (0.0%) | 0.002 |
| Unknown Gender | 10 (0.6%) | 10 (0.1%) | <0.001 | 0 (0.0%) | 10 (0.6%) | 0.002 |
| Hypertensive diseases | 1069 (61.7%) | 5834 (50.9%) | <0.001 | 1056 (61.5%) | 1069 (62.2%) | 0.648 |
| Neoplasms | 971 (56.1%) | 4000 (34.9%) | <0.001 | 957 (55.7%) | 957 (55.7%) | 1.000 |
| Hyperlipidemia, unspecified | 837 (48.3%) | 3678 (32.1%) | <0.001 | 823 (47.9%) | 822 (47.8%) | 0.973 |
| Overweight and obesity | 578 (33.4%) | 3112 (27.1%) | <0.001 | 571 (33.2%) | 562 (32.7%) | 0.744 |
| Diabetes mellitus | 499 (28.8%) | 2696 (23.5%) | <0.001 | 492 (28.6%) | 457 (26.6%) | 0.182 |
| Chronic kidney disease (CKD) | 430 (24.8%) | 2109 (18.4%) | <0.001 | 420 (24.4%) | 398 (23.2%) | 0.378 |
| Ischemic heart diseases | 414 (23.9%) | 1830 (16.0%) | <0.001 | 404 (23.5%) | 392 (22.8%) | 0.628 |
| Other chronic obstructive pulmonary disease | 271 (15.6%) | 1210 (10.6%) | <0.001 | 269 (15.7%) | 262 (15.3%) | 0.741 |
| Transplanted organ and tissue status | 109 (6.3%) | 349 (3.0%) | <0.001 | 102 (5.9%) | 77 (4.5%) | 0.055 |
| CD3+CD4+ (T4 helper) cells [#/volume] in Blood | 625 ± 385.89 | 576.31 ± 382.07 | <0.001 | 624.77 ± 385.49 | 588.59 ± 418.51 | 0.044 |
| At most 200 /uL | 409 (33.9%) | 1995 (34.3%) | <0.001 | 406 (33.9%) | 282 (33.4%) | <0.001 |
| BMI | 28.14 ± 6.27 | 28.68 ± 6.82 | 0.035 | 28.16 ± 6.3 | 28.67 ± 6.48 | 0.110 |
| At least 25 kg/m2 | 713 (41.2%) | 3359 (29.3%) | <0.001 | 702 (40.9%) | 676 (39.3%) | 0.365 |
| At least 30 kg/m2 | 415 (24.0%) | 2120 (18.5%) | <0.001 | 412 (24.0%) | 382 (22.2%) | 0.225 |
| HIV 1 RNA (viral load),  LOINC: 20447-9 | 12495.52 ± 61117.98 | 21299.48 ± 97274.2 | 0.070 | 12584.15 ± 61326.25 | 25203.58 ± 118450.86 | 0.051 |
| At most 50 {copies}/mL | 285 (67.1%) | 1702 (63.2%) | 0.081 | 282 (66.8%) | 304 (67.0%) | 0.318 |
| HIV 1 RNA (viral load),  LOINC: 70241-5 | 9718.84 ± 61644.18 | 20210.98 ± 104556.65 | 0.093 | 9763.62 ± 61938.29 | 22439.4 ± 158318.36 | 0.237 |
| At most 50 {copies}/mL | 263 (84.3%) | 747 (75.4%) | <0.001 | 261 (84.5%) | 94 (83.2%) | <0.001 |
| COVID-19 vaccine | 942 (54.4%) | 49 (0.4%) | <0.001 | 934 (54.4%) | 10 (0.6%) | <0.001 |

Supplemental Table 9. Patient counts for HIV medication between vaccinated and unvaccinated COVID-19 HIV positive cohorts, before and after propensity score matching.

|  | Before Matching | | | After Matching | | |
| --- | --- | --- | --- | --- | --- | --- |
| HIV Medication  Vaccinated: N = 1613 (93.1%)  Unvaccinated: N = 9316 (81.3%) | Vaccinated | Unvaccinated | p-Value | Vaccinated | Unvaccinated | p-Value |
| N | 1732 | 11463 |  | 1718 | 1718 |  |
| emtricitabine | 1217 (70.3%) | 6724 (58.7%) | <0.001 | 1207 (70.3%) | 1058 (61.6%) | <0.001 |
| Integrase inhibitors | 1008 (58.2%) | 5399 (47.1%) | <0.001 | 998 (58.1%) | 923 (53.7%) | 0.01 |
| tenofovir alafenamide | 1001 (57.8%) | 4949 (43.2%) | <0.001 | 994 (57.9%) | 803 (46.7%) | <0.001 |
| tenofovir disoproxil | 894 (51.6%) | 4768 (41.6%) | <0.001 | 886 (51.6%) | 803 (46.7%) | 0.005 |
| dolutegravir | 714 (41.2%) | 3634 (31.7%) | <0.001 | 707 (41.2%) | 634 (36.9%) | 0.011 |
| Protease inhibitors | 631 (36.4%) | 2967 (25.9%) | <0.001 | 625 (36.4%) | 512 (29.8%) | <0.001 |
| atorvastatin | 595 (34.4%) | 2577 (22.5%) | <0.001 | 584 (34.0%) | 545 (31.7%) | 0.157 |
| ritonavir | 544 (31.4%) | 2373 (20.7%) | <0.001 | 539 (31.4%) | 419 (24.4%) | <0.001 |
| lamivudine | 513 (29.6%) | 2789 (24.3%) | <0.001 | 509 (29.6%) | 482 (28.1%) | 0.309 |
| bictegravir | 503 (29.0%) | 2629 (22.9%) | <0.001 | 500 (29.1%) | 390 (22.7%) | <0.001 |
| cobicistat | 434 (25.1%) | 2456 (21.4%) | 0.001 | 432 (25.1%) | 397 (23.1%) | 0.163 |
| abacavir | 423 (24.4%) | 2190 (19.1%) | <0.001 | 420 (24.4%) | 388 (22.6%) | 0.198 |
| darunavir | 333 (19.2%) | 1714 (15.0%) | <0.001 | 330 (19.2%) | 300 (17.5%) | 0.186 |
| tenofovir | 317 (18.3%) | 1433 (12.5%) | <0.001 | 315 (18.3%) | 208 (12.1%) | <0.001 |
| efavirenz | 293 (16.9%) | 1597 (13.9%) | 0.001 | 291 (16.9%) | 300 (17.5%) | 0.684 |
| elvitegravir | 278 (16.1%) | 1722 (15.0%) | 0.266 | 276 (16.1%) | 284 (16.5%) | 0.712 |
| rosuvastatin | 252 (14.6%) | 891 (7.8%) | <0.001 | 246 (14.3%) | 192 (11.2%) | 0.006 |
| rilpivirine | 246 (14.2%) | 1208 (10.5%) | <0.001 | 246 (14.3%) | 184 (10.7%) | 0.001 |
| raltegravir | 245 (14.1%) | 1163 (10.1%) | <0.001 | 241 (14.0%) | 214 (12.5%) | 0.174 |
| pravastatin | 185 (10.7%) | 848 (7.4%) | <0.001 | 181 (10.5%) | 173 (10.1%) | 0.653 |
| atazanavir | 177 (10.2%) | 1002 (8.7%) | 0.044 | 176 (10.2%) | 167 (9.7%) | 0.609 |
| zidovudine | 112 (6.5%) | 677 (5.9%) | 0.359 | 112 (6.5%) | 105 (6.1%) | 0.623 |
| simvastatin | 71 (4.1%) | 415 (3.6%) | 0.324 | 69 (4.0%) | 96 (5.6%) | 0.031 |
| lopinavir | 70 (4.0%) | 418 (3.6%) | 0.417 | 69 (4.0%) | 65 (3.8%) | 0.724 |
| fosamprenavir | 21 (1.2%) | 108 (0.9%) | 0.287 | 20 (1.2%) | 10 (0.6%) | 0.067 |
| cabotegravir | 19 (1.1%) | 51 (0.4%) | <0.001 | 19 (1.1%) | 10 (0.6%) | 0.093 |
| lovastatin | 10 (0.6%) | 41 (0.4%) | 0.17 | 10 (0.6%) | 10 (0.6%) | 1.000 |
| pitavastatin | 10 (0.6%) | 45 (0.4%) | 0.266 | 10 (0.6%) | 10 (0.6%) | 1.000 |
| indinavir | 10 (0.6%) | 46 (0.4%) | 0.293 | 10 (0.6%) | 14 (0.8%) | 0.413 |
| saquinavir | 10 (0.6%) | 36 (0.3%) | 0.083 | 10 (0.6%) | 10 (0.6%) | 1.000 |
| tipranavir | 10 (0.6%) | 10 (0.1%) | <0.001 | 10 (0.6%) | 0 (0.0%) | 0.002 |
| fluvastatin | 0 (0.0%) | 10 (0.1%) | 0.219 | 0 (0.0%) | 10 (0.6%) | 0.002 |

Supplemental Table 10. PASC related outcomes between vaccinated and unvaccinated cohorts, before propensity score matching.

| Outcome | Vaccinated | Unvaccinated | OR (95% CI) | p-Value |
| --- | --- | --- | --- | --- |
| Mortality | 28 (1.6%) | 419 (3.6%) | 0.42 (0.29, 0.62) | 0.000 |
| Hypertension | 40 (5.9%) | 419 (7.5%) | 0.77 (0.55, 1.08) | 0.126 |
| Diabetes | 39 (3.1%) | 295 (3.4%) | 0.91 (0.65, 1.28) | 0.584 |
| Thyroid Disorders | 18 (1.1%) | 99 (0.9%) | 1.22 (0.74, 2.02) | 0.442 |
| Heart Disease | 47 (4.6%) | 483 (6.2%) | 0.72 (0.53, 0.97) | 0.033 |
| Malignancy | 36 (2.6%) | 214 (2.2%) | 1.21 (0.85, 1.73) | 0.294 |
| Thrombosis | 37 (2.6%) | 303 (3.1%) | 0.82 (0.58, 1.17) | 0.274 |
| Mental Disorders | 38 (6.8%) | 439 (8.7%) | 0.77 (0.55, 1.09) | 0.141 |
| Rheumatoid Arthritis | 10 (0.6%) | 21 (0.2%) | 3.1 (1.46, 6.59) | 0.002 |
| Respiratory Symptoms | 463 (25.8%) | 3013 (26.1%) | 0.99 (0.88, 1.1) | 0.811 |
| Headache | 161 (9.0%) | 1110 (9.6%) | 0.93 (0.78, 1.1) | 0.396 |
| Fatigue | 238 (13.3%) | 1530 (13.2%) | 1 (0.87, 1.16) | 0.976 |
| Bodyache | 78 (4.3%) | 542 (4.7%) | 0.92 (0.72, 1.18) | 0.521 |
|  |  |  |  |  |
| Diarrhea/constipation | 323 (18.0%) | 1860 (16.1%) | 1.14 (1, 1.3) | 0.042 |
| Cognitive Impairment | 20 (1.1%) | 121 (1.0%) | 1.07 (0.66, 1.71) | 0.794 |
| Disturbances to Smell and Taste | 10 (0.6%) | 66 (0.6%) | 0.98 (0.5, 1.9) | 0.943 |
| Fatigue | 180 (10.0%) | 1122 (9.7%) | 1.04 (0.88, 1.22) | 0.668 |
| Chronic cough | 232 (12.9%) | 1453 (12.6%) | 1.03 (0.89, 1.2) | 0.672 |
| Brain Fog | 51 (2.8%) | 276 (2.4%) | 1.2 (0.88, 1.62) | 0.247 |
| Palpitations | 55 (3.1%) | 362 (3.1%) | 0.98 (0.73, 1.3) | 0.879 |
| Chest pain | 261 (14.5%) | 1971 (17.1%) | 0.83 (0.72, 0.95) | 0.008 |
| Sexual desire or capacity | 10 (0.6%) | 34 (0.3%) | 1.9 (0.94, 3.85) | 0.070 |
| Dizziness | 132 (7.4%) | 796 (6.9%) | 1.07 (0.89, 1.3) | 0.467 |
| Gastrointestinal | 502 (28.0%) | 3375 (29.2%) | 0.94 (0.84, 1.05) | 0.287 |
| Hair loss | 22 (1.2%) | 129 (1.1%) | 1.1 (0.7, 1.73) | 0.682 |
| Abnormal movements | 53 (3.0%) | 276 (2.4%) | 1.24 (0.92, 1.68) | 0.151 |
